# Does Islet Size Really Influence Graft Function After Clinical Islet Transplantation?

**DOI:** 10.1097/TP.0000000000002392

**Published:** 2018-10-26

**Authors:** Stephen J. Hughes, Paul A. Bateman, Sarah E. Cross, Daniel Brandhorst, Heide Brandhorst, Ioannis Spiliotis, Chitrabhanu Ballav, Miranda Rosenthal, Martin K. Rutter, James Shaw, Stephen Gough, Paul R.V. Johnson

**Affiliations:** 1 Islet Transplant Research Group, Nuffield Department of Surgical Sciences and Oxford Centre for Diabetes, Endocrinology and Metabolism (OCDEM), University of Oxford, Oxford, United Kingdom.; 2 Radcliffe Department of Medicine, OCDEM, University of Oxford, Oxford, United Kingdom.; 3 UCL Institute of Immunity and Transplantation, Royal Free Hospital, London, United Kingdom.; 4 Division of Diabetes, Endocrinology and Gastroenterology, School of Medical Sciences, Faculty of Biology, Medicine and Health, University of Manchester, Manchester, United Kingdom.; 5 Manchester Diabetes Centre, Central Manchester University Hospitals NHS Foundation Trust, Manchester Academic Health Science Centre, Manchester, United Kingdom.; 6 Institute of Cellular Medicine, Newcastle University, Newcastle upon Tyne, United Kingdom.

## Abstract

**Background:**

It has been proposed that islet transplants comprised primarily of small rather than large islets may provide better graft function, due to their lower susceptibility to hypoxic damage. Our aim was to determine whether islet size correlated with in vivo graft function in islet transplant recipients with C peptide–negative type 1 diabetes when islets have undergone pretransplant islet culture.

**Methods:**

Human pancreatic islets were isolated, cultured for 24 hours and infused by standardized protocols. Ninety-minute stimulated C-peptide concentrations were determined during a standard meal tolerance test 3 months posttransplant. The islet isolation index (IEq/islet number) was determined immediately after isolation and again before transplantation (after tissue culture). This was correlated with patient insulin requirement or stimulated C-peptide.

**Results:**

Changes in insulin requirement did not significantly correlate with islet isolation index. Stimulated C-peptide correlated weakly with IEq at isolation (*P* = 0.40) and significantly with IEq at transplantation (*P* = 0.018). Stimulated C-peptide correlated with islet number at isolation (*P* = 0.013) and more strongly with the islet number at transplantation (*P* = 0.001). In contrast, the correlation of stimulated C-peptide and islet isolation index was weaker (*P* = 0.018), and this was poorer at transplantation (*P* = 0.034). Using linear regression, the strongest association with graft function was islet number (*r* = 0.722, *P* = 0.001). Islet size was not related to graft function after adjusting for islet volume or number.

**Conclusions:**

These data show no clear correlation between islet isolation index and graft function; both small and large islets are suitable for transplantation, provided the islets have survived a short culture period postisolation.

## Abstract 2

By analyzing the insulin requirements from 25 islet transplantation recipients, Hughes et al determined the strongest association with graft function was islet number while islet size was not related to graft function after adjusting for islet volume or number.

Clinical islet transplantation is an effective treatment for stabilizing glycemic control in patients with type 1 diabetes complicated by severe hypoglycemia. Unfortunately, numerous donor and retrieval factors, such as donor age, body mass index (BMI), cold ischemia time, collagenase lot, organ perfusion quality, or retrieval team can influence the outcome of the islet isolation process and affect islet yields,^1-6^ whereas factors such as donor age have been shown to be important determinants of islet graft function.^5,7^ As a result, at best in leading clinical centers worldwide, only 50% of islet preparations are of sufficient quality and high enough yield for transplantation.^2,3,8^ In addition, early posttransplant islet destruction, variable engraftment, the nature of the immunosuppression used, and subsequent graft deterioration over time all contribute to variable long-term islet function.^9-12^ Indeed, it is widely accepted that less than 50% of transplanted islets survive the initial stage of engraftment.^13-16^ Consequently, there has been considerable debate about the optimal composition of islet preparations used for transplantation. It has been long been established that total islet graft volume and islet number are critical for transplantation success,^17-19^ but more recently, it has also been suggested that grafts comprising predominantly of small islets may be preferential due to their reduced susceptibility to hypoxia and central necrosis. Indeed, a number of in vitro studies have shown that smaller islets have superior function compared with larger ones.^20-24^ However, data confirming this in clinical studies are more limited. Lehmann et al^15^ acknowledged the importance of the factors discussed above for islet success and transplantation outcomes but also reported that better function was associated with grafts comprising of smaller islets in simultaneous islet-kidney transplant recipients; however, there were only 7 recipients in the study, all in end-stage renal failure. Importantly, the islets used within that study were transplanted “fresh” after islet isolation, rather than having the benefit of undergoing a period of islet culture. A larger study in autologous islet recipients after pancreatectomy also supported this conclusion, although in this study, grafts of marginal islet mass were often transplanted.^25^ Our own observation,^26^ however, is that provided larger islets survive a period of pretransplant islet culture, they can confer advantages over smaller islets in terms of graft function and graft longevity. To investigate this discrepancy, we aimed to correlate islet size (preculture and postculture) with in vivo measures of graft function in all islet transplant recipients receiving their first transplant in our islet transplant program over a 6-year period.

## METHODS

Human pancreases were retrieved with appropriate consent and ethical approval from 25 deceased multiorgan donors (15 women and 10 men). Median (range) donor age was 49 years (34-60 years) and median donor BMI of 29 kg/m^2^ (23-37 kg/m^2^). After standardized procurement, the pancreas was transported in University of Wisconsin solution at 4°C to the Diabetes Research & Wellness Foundation Human Islet Isolation Facility in Oxford. Median (range) cold ischemia time was 6.7 hours (4-10.5 hours). Pancreatic islets were isolated using a standard protocol as described previously.^27^ After infusion and digestion with collagenase NB 1 and neutral protease NB enzyme blend (Serva, Heidelberg, Germany), islets were purified using a Ficoll-based continuous density gradient and quality-assessed (for sterility, viability, purity, and yield) as previously described.^28-30^ Islet number and size were determined in dithizone stained islet samples by visual microscopic inspection by comparing the stained islet- particles in multiple representative samples against a size graticule ranging from 50 to greater than 400 μM in size. Each preparation was resuspended in 150- to 200-mL CMRL-based culture media and multiple samples collected whilst in continuous suspension. The islets in the samples were visualized by dithizone staining under a light microscope and the size of each islet in the sample determined by comparison with a calibrated graticule in the microscope objective; the total number of islets in each sample and their sizes were recorded. Islet preparations were then cultured for a minimum of 24 hours in the CMRL media in a humidified atmosphere at 37°C before a full reassessment. Islets were then transplanted either locally or at a satellite transplant center. If allocated to a recipient at a satellite transplant center,^31^ the islet preparation was transported by road (maximum journey time, 8 hours) in cooled standard 500 mL blood transfusion bags with a temperature monitor.^27,28^ After confirmation of satisfactory temperature maintenance during transport together with confirmation of maintained islet integrity and viability from a side-arm islet sample, islets were transplanted directly from the transport bag.

Only recipients undergoing their first islet transplant were included in this study (n = 25). In all cases, islets were transplanted by percutaneous transhepatic delivery into the portal vein under radiological visualization. All patients received a minimum of 5000 Islet Equivalents (IEq) per kg body weight (median, 5500 IEq/kg). Peritransplant, recipients were placed on an intravenous glucose/insulin sliding scale and received a heparin infusion according to published protocols.^32^

Each patient received a standard immunosuppression protocol comprising alemtuzumab (Campath) induction 30 mg before and on day 1 after transplant; tacrolimus at 0.05 mg/kg per day titrated to a serum trough level of 8 to 12 ng/mL; mycophenolate mofetil (500 mg BD) and total daily insulin requirements were monitored at routine intervals. Intensified insulin regimens were continued in all postdischarge, with the goal being maintenance of optimal glycemic control.

In a subgroup of the recipients (n = 18), 90-minute stimulated C-peptide concentrations were determined during a standard meal tolerance test 3 months posttransplant. Grafts with primary nonfunction as defined by a stimulated C-peptide level less than 50 pmol/L were excluded from all subsequent analysis. Assessment of stimulated C- peptide was made at 3 months posttransplantation as an indicator of early graft function. It was also done at this time to avoid any complicating effects of a subsequent second transplant which is carried out in the UK islet transplant program.^31^ The beta score was also determined for each transplant recipient at 3 months posttransplant as described by Ryan et al.^33^

For each islet preparation transplanted, the islet isolation index (IEq/islet number) was calculated; the total IEq of the preparation is the internationally agreed standard for the total volume of the islet graft. The islet isolation index therefore is a measure of the average size of each islet within the graft, with an index of 1 indicating an average islet size of 150 μM in diameter. Islet isolation index was related to 3 variables of graft function in transplant recipients: (a) the change in insulin requirement (before vs 3 months after transplant), (b) the 90-minute stimulated C-peptide level taken 3 months posttransplant, and (c) the beta score at 3 months posttransplant.^33^

For statistical analysis, data were tested for normalcy and are presented as means ± standard deviations (SD) or median (range) depending on data distribution. Groups were compared using paired Student *t* test when appropriate. Correlations were assessed using Pearson correlation coefficient. Linear regression was performed with preculture and postculture islet isolation variables (age, BMI, CIT, viability, purity, IEQ/kg, Islet number/ kg, and islet isolation index) as exposure variables and change in insulin dose or stimulated C-peptide as outcome variables. Multiple regression was conducted to assess the impact of islet isolation outcomes on stimulated C-peptide, adjusting for donor age and BMI, or on beta score, adjusting for donor age and prep purity. Final multiple regression models were fitted using backward stepwise elimination of candidate variables. *P* less than 0.05 was considered statistically significant. Finally, a power calculation was carried out assuming *P* less than 0.05, a sample size of 25, and α = 0.05. To obtain an *R* value of 0.5, the power was 0.73; *R* value of 0.6, power was 0.9; and an *R* value of 0.7, the power was 0.98. Data analysis was performed using SPSS, Version 20 & 24 (2011) (IBM Corp, Armonk, NY).

## RESULTS

Donor and islet preparation characteristics are summarized in Table 1. Immediately postisolation, the islet yield (mean ± SD) was 454 800 ± 190 900 IEq with a mean islet number of 232 320 ± 114 060 islets, purity ranged from 50% to 90% and viability was 75% or greater in all cases. After 24 hours in culture, the islet number had significantly reduced to 197 300 ± 91 200 islets (*P* = 0.013, paired *t* test) whereas the small reduction in IEq to 408 600 ± 126 700 was not significant. Before transplantation, patients required a mean of 31.5 ± 13.4 U insulin per day (0.48 ± 0.18 U/kg body wt per day), which was reduced by 17.1 ± 9.9 U/d to 15.3 ± 14.0 U/d (0.22 ± 0.18 U/ kg body wt per day) at 3 months posttransplant. The mean stimulated C-peptide concentration during a meal tolerance test at 3 months posttransplant was 624 ± 524 pmol/L (Table 1).

**TABLE 1:**
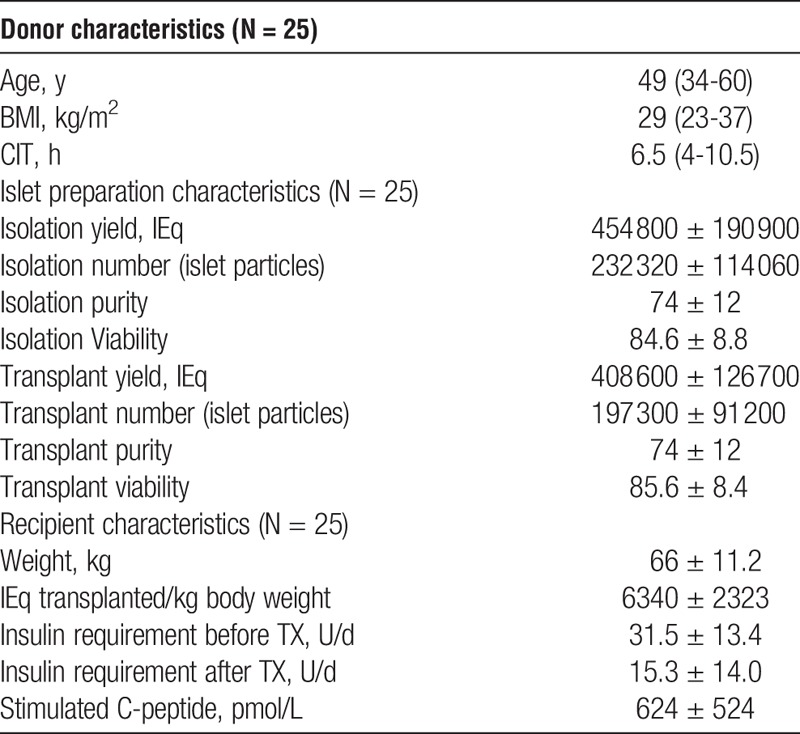
Donor and recipient characteristics and islet preparation data. Data are presented as mean ± SD except for donor characteristics which are median (range)

**FIGURE 1:**
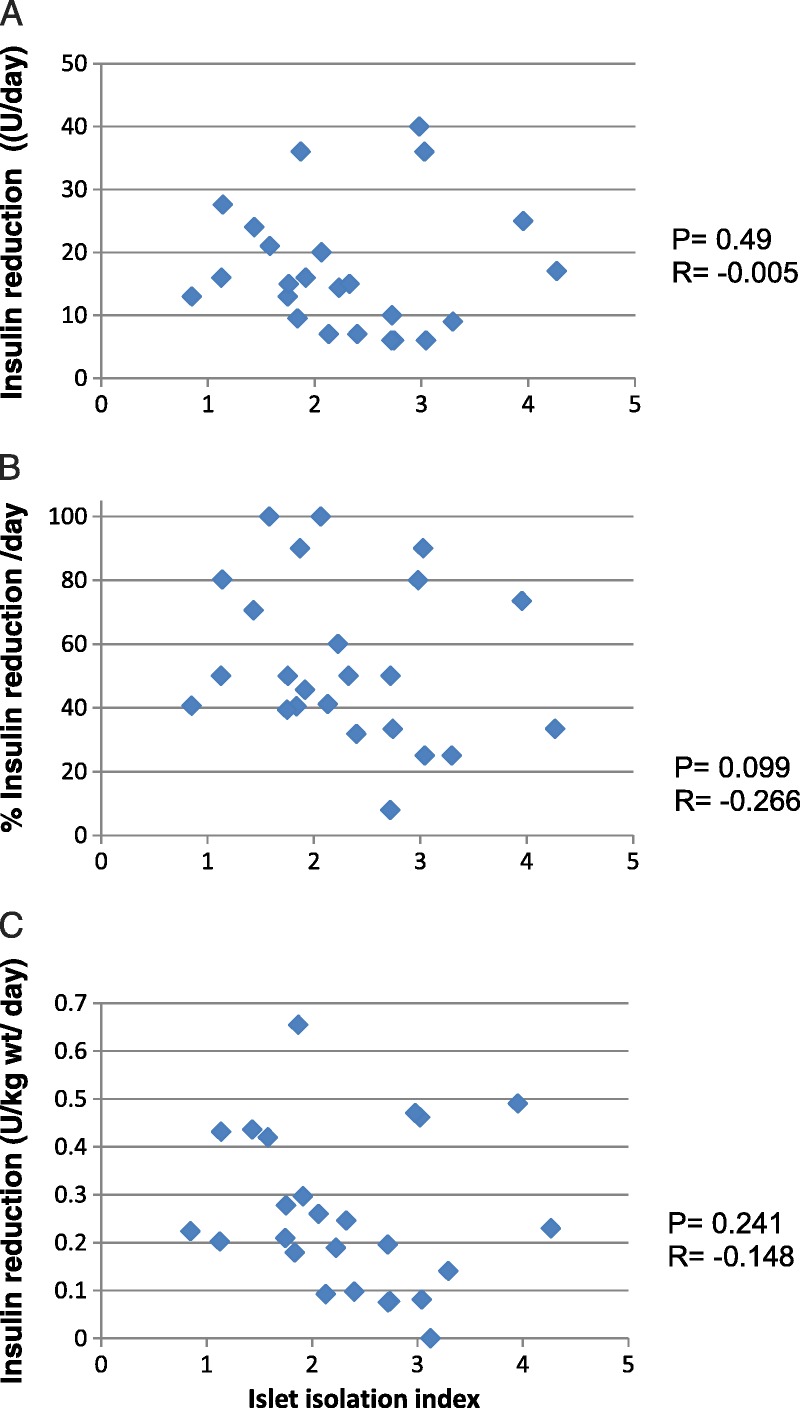
Change in insulin requirement per day (A) or % change /day (B) or per kg body weight /day (C) in relation to islet isolation index of the islet graft.

The change in insulin requirement before and 3 months after transplant was correlated with islet size as assessed by the islet isolation index of the transplanted islet preparation (Figure 1). There was no significant correlation with changing islet isolation index of the transplanted islets and graft function; either absolute change in insulin requirement (*r* = −0.005, *P* = 0.49), percentage change in insulin requirement (*r* = −0.27, *P* = 0.099), or insulin change per kg body weight of the recipient (*r* = −0.15, *P* = 0.24). The change in insulin requirement also correlated poorly with IEQ (*r* = 0.11, *P* = 0.49) or IEQ/kg body weight (r = 0.05, *P* = 0.4) (data not shown).

**FIGURE 2:**
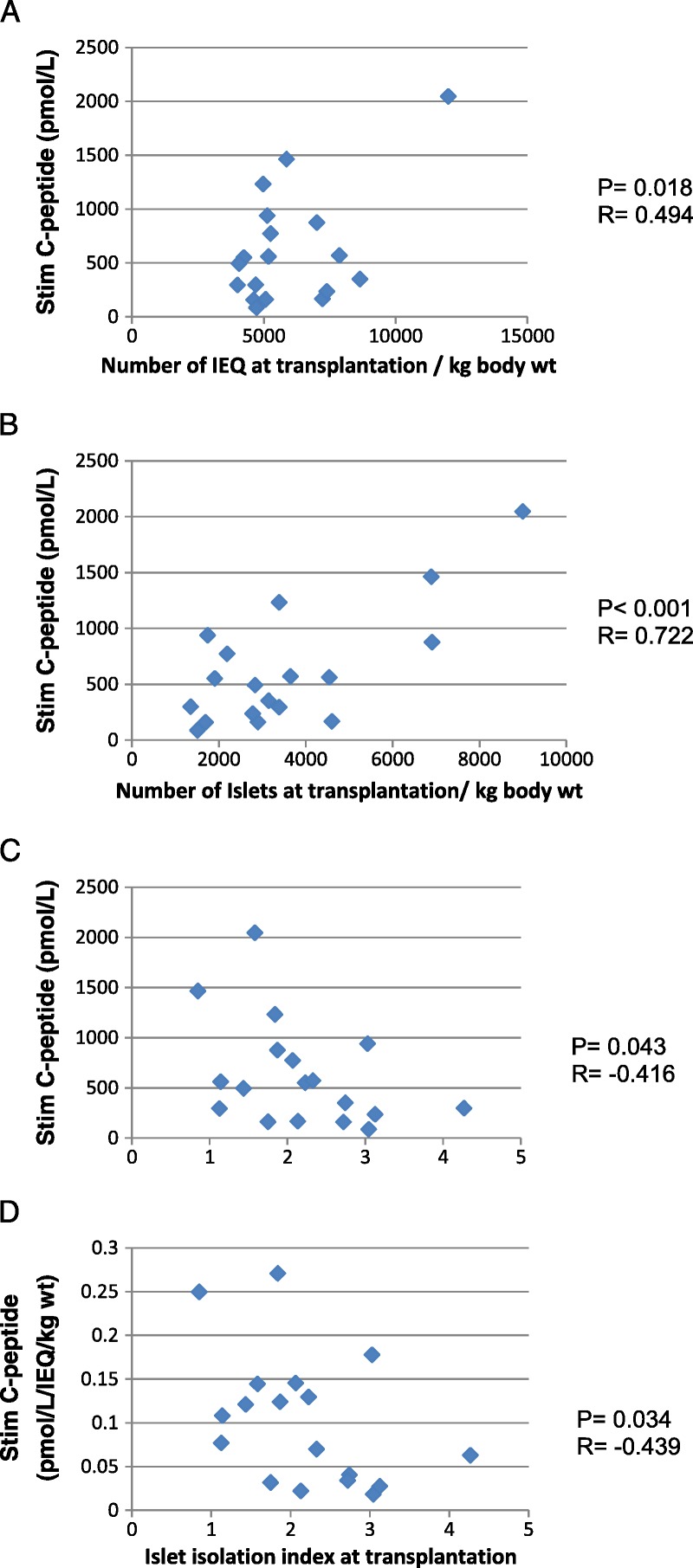
Correlation of stimulated C-peptide with the number IEQ (A), islets (islet particles) (B), or islet isolation index (C) or stimulate C-peptide/kg wt (D) at.transplantation.

**FIGURE 3:**
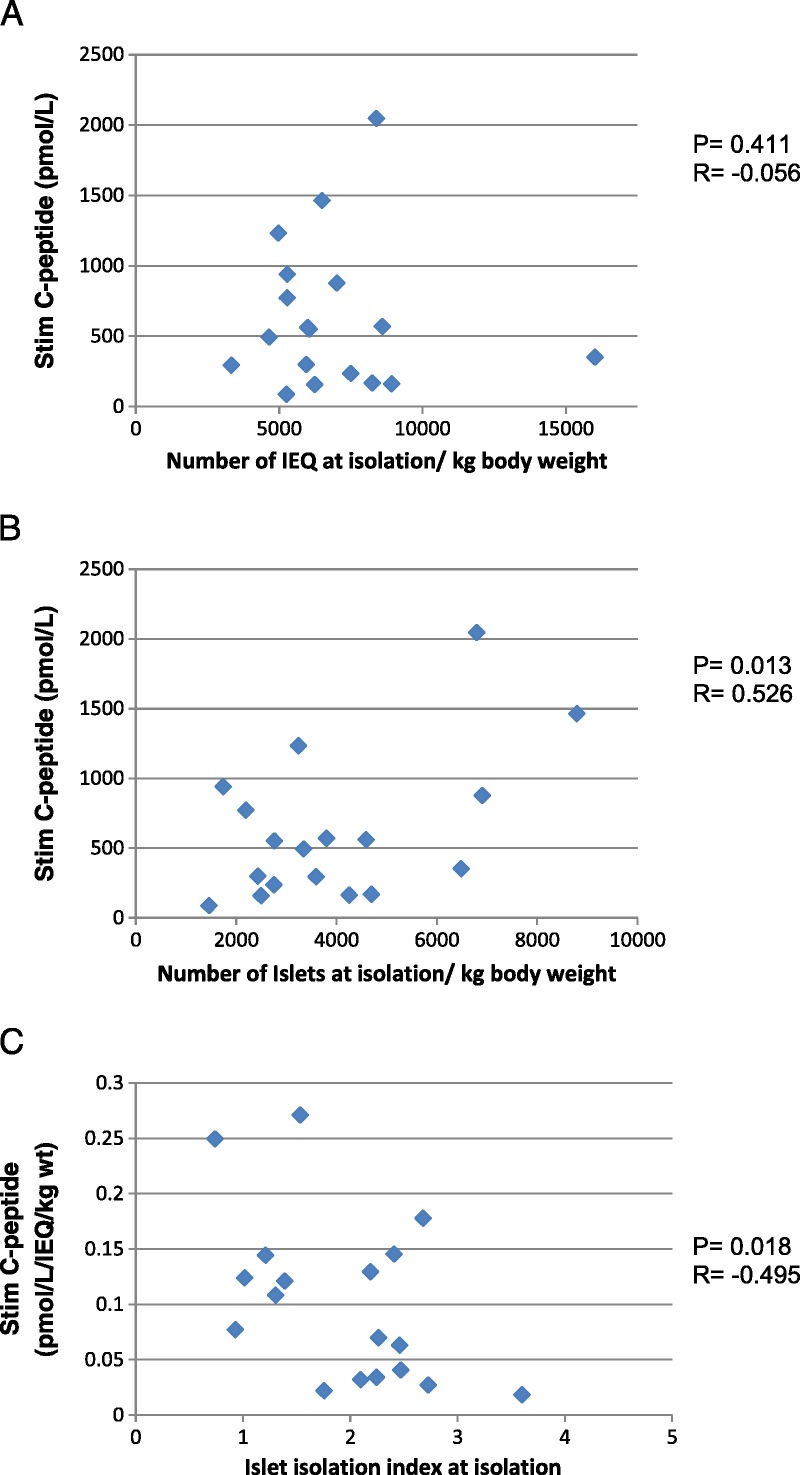
Correlation of stimulated C-peptide with the number of IEQ (A), number of islets (islet particles (B) or islet isolation index (C) at isolation.

In contrast, stimulated C-peptide levels at 90 minutes during a meal tolerance test were significantly correlated with the number of islets in the graft (*P* = 0.004, *r* = 0.605), and the strongest correlation determined was with the number if islets in the graft per kg body weight (*r* = 0.722, *P* < 0.001, Figure 2B). In contrast, the correlations with IEq (r = 0.17, *P* = 0.25) or IEq per kg recipient body weight (*r* = 0.494, *P* = 0.018) were poorer (Figure 2A). When plotted against the islet isolation index of the transplanted islets, the correlation with stimulated C-peptide remained significant (*r* = −0.416, *P* = 0.043) as did stimulated C-peptide per IEq per kg body weight of the recipient (*r* = −0.439; *P* = 0.034, Figures 2C and D). As the islet yield and number had changed during culture from the values measured at isolation, we also determined correlations for stimulated C-peptide with parameters measured immediately after isolation. The correlation of stimulated C-peptide with islet number was significant (Figure 3B, *r* = 0.526; *P* = 0.013) but poorer than that determined at transplantation, whereas the correlation with IEq at isolation was weak (Figure 3A, *r* = −0.056; *P* = 0.411). The correlation of stimulated C-peptide per IEQ per kg body weight, however, was more significant with the islet isolation index of the preparations determined immediately after isolation (Figure 3C, *r* = −0.495; *P* = 0.018) than that determined at transplantation.

We assessed the strength of univariable- and multivariable-adjusted associations between islet isolation yield parameters, purity, viability, donor variables (age, BMI, CIT), and 90-minute stimulated C-peptide levels using linear regression. These data confirmed the observation that the factor most strongly associated with graft function in univariable analysis was the number of islets in the graft (*P* = 0.001; Table 2). In a model including both total islet volume (IEq/kg) and islet size (Isolation index; IEq/ Islet number), total islet volume and donor age were related to 90-minute stimulated C-peptide levels (islet volume, β =0.101, *P* = 0.019; donor age β = −26.0, *P* = 0.034, Table 2). In a model including both islet number (number/kg) and islet size, only the islet number was related to the 90-minute stimulated C-peptide levels (β = 0.19, *P* = 0.001; Table 2). Islet size was not related to graft function after adjusting for total islet volume or islet number or any of the other variables analyzed.

**TABLE 2:**
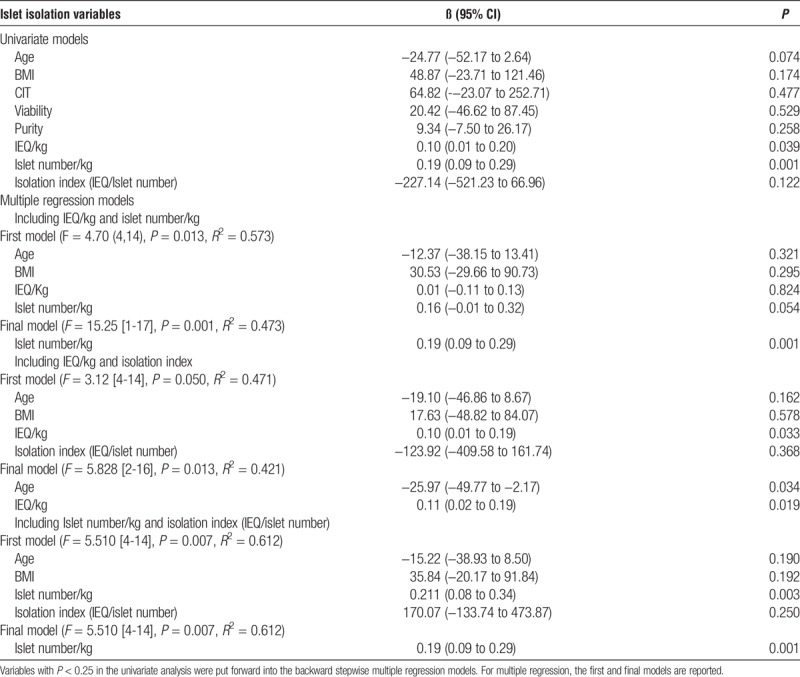
Univariate and multivariate relationships between islet isolation variable and 90-minute stimulated C-peptide levels at 3 months

Islet isolation index, islet volume, and islet number at transplantation were also correlated with the beta score as another measure of graft function at 3 months posttransplant (data not shown). The correlation of the beta score with islet isolation index at transplantation (r = -0.584, *P* = 0.011) was poorer than that with islet number (r = 0.784, *P* = 0.001). Univariate linear regression with the following factors: islet isolation variables and donor variables with beta score showed that islet number/ kg (*P* = 0.026), islet isolation index (*P* = 0.022), and islet purity significantly (*P* = 0.027) affected this measure of graft function. Further multiple regression analysis, however, indicated that both islet number/kg (*P* = 0.032) and islet purity (*P* = 0.38) but not islet isolation index had a significant effect in the final model of the analysis.

## DISCUSSION

In this study, we tested the existing hypothesis that islet grafts comprising predominantly smaller islets have superior function compared with those composed of large islets. We have done this by correlating islet size within transplanted grafts with the posttransplant metabolic outcomes of the recipients. Several studies using experimental animal models have previously shown that smaller islets are potentially more beneficial as islet grafts. Smaller islets have been shown to have improved nutrient supply, with larger islets depleted of both oxygen and glucose at the core.^34,35^ Smaller islets also exhibit improved insulin secretory function in vitro, exhibit a higher vascular density, and function preferentially in transplant models.^20-24^ Studies with human islets in vitro also reproduce some of these results; small human islets were shown to be less susceptible to hypoxia and had improved secretory function compared with large islets.^15,36^

However, studies investigating whether the hypothesis is born out in allotransplant recipients of islet grafts have been limited. Lehmann et al^15^ correlated islet size with stimulated C-peptide in 7 simultaneous islet-kidney transplant recipients with type 1 diabetes. This study showed that increasing the number of islets in the graft significantly correlated with graft function and critically, the most significant correlation was found between islet isolation index and stimulated C-peptide per kg body weight of the recipient. The authors argued that correction for islet size proved to be the best predictor of graft function with 89% of the variability (as indicated by the correlation *R*^2^) being accounted for by the islet isolation index parameter. However, the authors^15^ recognized that their findings should be confirmed in a larger series of islet transplants. The present study was undertaken to do this. Here, we report on 2 parameters of islet function in a larger cohort of islet transplant recipients; reduction in insulin requirement per day and stimulated C-peptide levels during a meal tolerance test. Neither of the parameters analyzed show convincing correlation with the islet isolation index. There was no significant correlation with changes in patient insulin requirement in absolute terms or per kg body weight or when expressed as % change. Nor did the present study reproduce the findings of Lehmann et al,^15^ as the most significant correlation we found was between the number of islets in the graft and graft function as measured by stimulated C-peptide. When the correction for islet size was included, the correlation was poorer and *R*^2^ reduced with only 19.3% of the variability accounted for by this parameter.

Two factors may contribute to the different findings in our study compared with those of Lehmann et al.^15^ First, their study was in patients undergoing simultaneous islet kidney transplants, whereas all our patients received islet transplants alone. Second, their observations were made using islets that had not undergone the benefit of a period of pretransplant islet culture.^37^ In the UK islet transplant program, all islet preparations considered for transplantation undergo a minimum of 24-hour culture before transplant.^27^ Using this protocol, any marginal grafts susceptible to hypoxic damage and leading to declining viability are screened out and therefore do not proceed to transplantation. When islets are transplanted immediately after isolation or where only the immediate postisolation characteristics (of yield and viability) are used to decide suitability for transplantation, declining or marginal grafts are potentially transplanted. Because large islets are particularly susceptible to central necrosis, it can be postulated that when islets are transplanted immediately after isolation, it is the large islets that are particularly vulnerable to destruction, whereas large islets that are transplanted after surviving a period of islet culture are primarily robust and fully viable. Furthermore, the culture process itself may promote an adaptive or remodeling process in the islet preparation in which susceptible islets are selectively lost. Changes in islet morphology (rounding up) during culture and the significant reduction in islet number after culture in the present study is evidence of just such a process. We have also recently shown that short-term tissue culture ameliorates the destruction of human islets by instant blood-mediated inflammatory reaction.^38^ Thus it is possible that islets cultured for short periods before transplant are effectively preconditioned and any correlation between islet size and function in grafts is lost or reduced. In the present study, the correlation of islet isolation index and graft function was stronger in freshly isolated islets compared with islets cultured for a minimum of 24 hours at transplant.

In addition, it may be the case that there are too many other confounding donor and recipient factors which override any effect of islet size in determining graft function.^25^ Suszynski et al^25^ argued that islet autotransplant patients offered a good model for studying the effect of the size of islets in the graft as these patients are uncompromised by factors, such as autoimmunity, prolonged diabetic environment,^39^ and possibly IBMIR.^16,40^ In a cohort of 58 patients receiving islet autotransplants after pancreatectomy for chronic pancreatitis, the islet isolation index correlated with the change in insulin requirement and insulin independence rates in the patients.^25^

Although the number of observations we present here is significantly (threefold) greater that those reported by Lehmann et al,^15^ our analysis indicates that this retrospective study is still underpowered. Unfortunately, the number of patients available to study has been limited by the practice in the UK islet transplant program to retransplant islet recipients within 6 months of receipt of the first graft.^31^ The effect of a second islet graft to potentially change the mean islet size transplanted in the recipient makes the analysis carried out in the present study impossible.

Another theoretical limitation of the present study is that the method used to determine islet size in the graft samples may be less accurate than computational methods^37^ which may capture more of the population of smaller islets during analysis. In the present study, islet sizes were determined by inspection and comparison using a graticule after microscopic visualization. Our methodology has been agreed and validated between the participating islet isolation laboratories operating within the UK islet transplant program. However, there is no evidence of a significant difference in the size distribution of islets in our study compared with the study by Lehmann et al^15^ because the range of islet isolation indices here (0.8-4.2) overlap almost completely with those which they reported (0.75-3.3).^15^

Finally, it is important to note that changes in insulin requirement as a measure of graft function are less robust than stimulated C-peptide measurements. The latter is a positive output directly measuring graft function, whereas changes in insulin requirement are indirect and may not be necessarily linearly responsive to graft size. This less objective parameter, however, has been used as the indicator of islet graft function in previous studies on islet transplant recipients.^25,41^

In summary, our study found no clear correlation between islet isolation index and islet graft function in recipients receiving islets that had been cultured before implantation. These data therefore do not support the hypothesis that smaller islets have better function when used in clinical islet transplantation; large islets are equally suitable provided they have undergone and survived a short period of culture postisolation.
